# Discovery of novel, non-acidic mPGES-1 inhibitors by virtual screening with a multistep protocol

**DOI:** 10.1016/j.bmc.2015.05.045

**Published:** 2015-08-01

**Authors:** Stefan M. Noha, Katrin Fischer, Andreas Koeberle, Ulrike Garscha, Oliver Werz, Daniela Schuster

**Affiliations:** a Computer Aided Molecular Design (CAMD) Group, Institute of Pharmacy/Pharmaceutical Chemistry, University of Innsbruck, Innrain 80/82, A-6020 Innsbruck, Austria; b Chair of Pharmaceutical/Medicinal Chemistry, Institute of Pharmacy, University of Jena, Philosophenweg 14, D-07743 Jena, Germany

**Keywords:** Inflammation, mPGES-1, Virtual screening, 3D pharmacophore, Kruskal–Wallis test

## Abstract

Microsomal prostaglandin E_2_ synthase-1 (mPGES-1) inhibitors are considered as potential therapeutic agents for the treatment of inflammatory pain and certain types of cancer. So far, several series of acidic as well as non-acidic inhibitors of mPGES-1 have been discovered. Acidic inhibitors, however, may have issues, such as loss of potency in human whole blood and in vivo, stressing the importance of the design and identification of novel, non-acidic chemical scaffolds of mPGES-1 inhibitors. Using a multistep virtual screening protocol, the Vitas-M compound library (∼1.3 million entries) was filtered and 16 predicted compounds were experimentally evaluated in a biological assay in vitro. This approach yielded two molecules active in the low micromolar range (IC_50_ values: 4.5 and 3.8 μM, respectively).

## Graphical abstract

**Figure:**
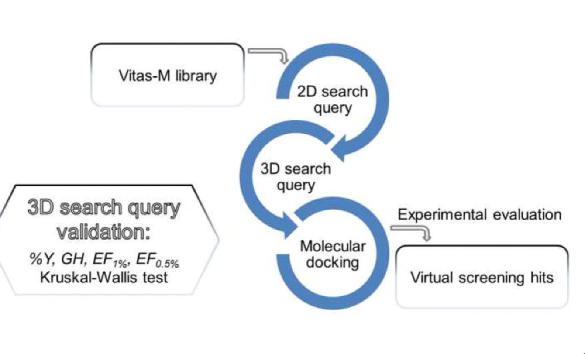

## 1 Introduction

In the arachidonic acid cascade, the activity of the cytosolic phospholipase A_2_ is required for the release of arachidonic acid (AA), a critical precursor molecule for pro-inflammatory mediators. Different enzymatic pathways convert AA into distinct eicosanoids. These mediators regulate various physiological processes and also trigger multiple effects in various human diseases.^1,2^ Among these mediators, prostaglandin E_2_ (PGE_2_) is well recognized as critical bioactive molecule. High PGE_2_ levels, typically occurring in inflammation, are relevant for swelling, fever, and inflammatory pain, and thus, pharmacological inhibition of PGE_2_ biosynthesis is considered a promising opportunity for the treatment of inflammatory pain, for example, in rheumatic diseases.^3^ Additionally, PGE_2_ synthesis is important in tumor growth and cancer progression.^4–6^ PGE_2_ is produced from the cyclooxygenase (COX)-derived prostaglandin H_2_ (PGH_2_) by PGE_2_ synthases (PGES) (EC 5.3.99.3).^7^ Among the three PGES isoenzymes, the microsomal PGES-1 (mPGES-1) displays a unique role because its expression is induced in the inflammatory response, similar to COX-2.^8^

By inhibiting mPGES-1 as the terminal synthase in PGE_2_ biosynthesis, mPGES-1 inhibitors are considered very promising regarding their side effect profile.^9,10^ The application of other anti-inflammatory agents, such as unspecific COX inhibitors, traditional nonsteroidal anti-inflammatory drugs (NSAIDs), or COX-2-selective inhibitors (coxibs), is associated with side effects concerning, among others, the renal function and effects on the gastrointestinal tract.^11^ In contrast, during prolonged inhibition of mPGES-1 in dogs, pronounced effects on the renal function were not observed.^12^ So far, there is no mPGES-1 inhibitor available for clinical use, although data from pre-clinical studies stressed the relevance of mPGES-1 inhibitors as potentially therapeutic agents. Therefore, the development of mPGES-1 inhibitors is highly relevant.^13^

**Figure 1:**
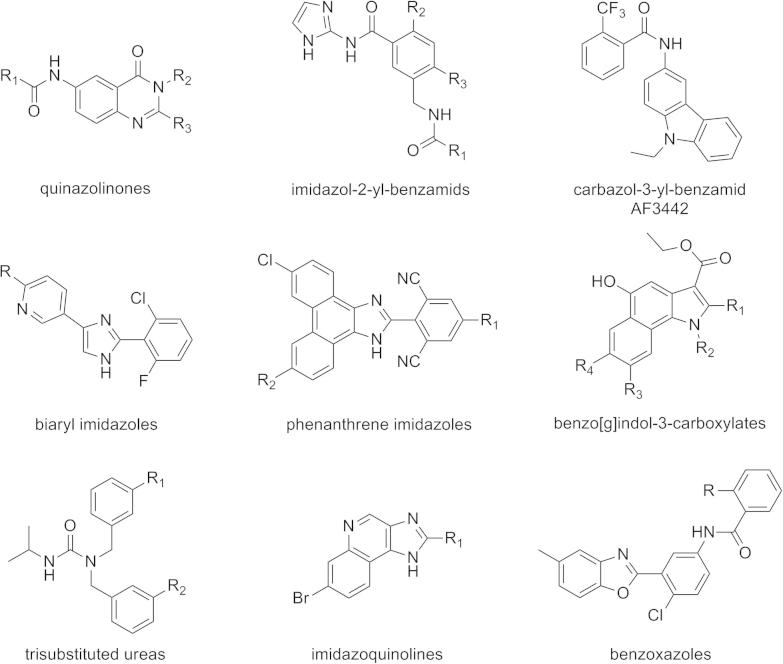
Chemical series of non-acidic mPGES-1 inhibitors are depicted with 2D structures.

A series of mPGES-1 inhibitors is reported in the literature, of which several comprise an acidic functionality, such as an oxicam template,^14^ a sulfonamide group^15^ or a carboxylic acid moiety.^16,17^ Unfortunately, acidic molecules suppressing mPGES-1 activity may have inferior potency in human whole blood seemingly due to unspecific plasma protein binding.^14,16^ This suggests that the design and identification of novel, non-acidic chemical scaffolds is warranted. As an overview, non-acidic chemical scaffolds of mPGES-1 inhibitors, which were reported so far, are shown in Figure 1.^18–27^

Previously, we reported the discovery of acidic mPGES-1 inhibitors using a pharmacophore-based virtual screening approach. Using this screening protocol, acidic inhibitors from synthetic libraries were discovered. The most potent inhibitors exhibited IC_50_ values in the sub-micromolar range.^17^ Additionally, mPGES-1 inhibitors with comparable potency from *Lichen* species were discovered using the previously reported pharmacophore model.^28^ Comparably, other groups have reported virtual screening approaches to find novel mPGES-1 inhibitors. For instance, Rörsch et al. applied a multistep ligand-based virtual screening protocol to discover novel and non-acidic mPGES-1 inhibitors.^29^ In addition, several active compounds were discovered by applying docking-based screening strategies, of which some even elicited high potency.^30–33^ Docking-based virtual screening campaigns towards mPGES-1 have been facilitated as a 3D electron crystallography structure was reported in 2008.^34^ In 2013, a high-resolution X-ray crystal structure of mPGES-1 has been resolved.^35^ Very recently an X-ray crystal structure of mPGES-1 with a co-crystallized ligand has been reported.^36^

In this study, a novel concept for the validation of the 3D pharmacophore model was applied using the Kruskal–Wallis test.^37^ This test was suggested as a robust investigation of the discriminatory power of distinct virtual screening methods, and was previously used for the comparative assessment of docking and scoring functions.^38,39^ The analysis with the Kruskal–Wallis test is characterized as less artifact-prone and also enables a *post hoc* test, rendering this analysis an attractive method in the validation also for pharmacophore-based virtual screening.^38,39^

## 2 Materials and methods

### 2.1 Study design

**Figure 2:**
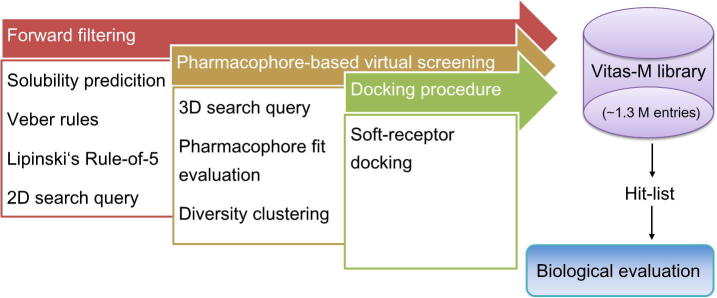
Overview of the virtual screening protocol.

In brief, we consecutively performed forward filtering, using 2D similarity screening, and pharmacophore-based virtual screening. The most interesting molecules which were retained thereof, accounting in addition pharmacophore fit evaluation and diversity clustering, were submitted to molecular docking. Finally, this protocol was applied to prospective virtual screening of the Vitas-M library (http://www.vitasmlab.com/). The hit-list was visually inspected to select compounds for a biological evaluation to discover novel and non-acidic mPGES-1 inhibitors (Fig. 2).

### 2.2 Software specifications

The computational studies were performed on a workstation running Microsoft Windows 7, which was employed for the work with the molecular modeling package Discovery Studio version 3.5^40^ and PipelinePilot 8.0.1.^41^ In parallel, the calculations for the work with Maestro suite 9.2.112^42^ were performed on a workstation running OpenSuse 12.1. The statistical evaluation was performed within Microsoft Excel 2010 and its add-in Analyse-it Method Evaluation version 2.26.^43^

### 2.3 Validation

#### 2.3.1 Concept

We assessed the discriminatory power of the 3D pharmacophore model by following the workflow reported by Seifert et al.^38,39^ In this work, the discriminatory power of docking and scoring functions was assessed by ANOVA (analysis of variance) or a nonparametric version of it, that is, the Kruskal–Wallis test.^37^ Because this concept can also be useful for the development of 3D pharmacophore models, this analysis was included in the model validation and conducted as an extension to the validation with benchmarking experiments. So a validation set, set_1, was assembled and used for screening experiments with the hypotheses. The statistical evaluation of the results was accomplished with the Kruskal–Wallis test and a *post hoc* test. Furthermore, benchmarking experiments were conducted by screening a second validation set, set_2, and calculating well-established performance metrics.

#### 2.3.2 Validation sets and calculations

Set_1 comprised highly active (IC_50_ ⩽0.5 μM), medium active (IC_50_: 0.5–5 μM), and confirmed inactive molecules (IC_50_ >5 μM) from several congeneric series of non-acidic mPGES-1 inhibitors, with 14 molecules in each group. It consisted, in total, of 42 molecules. For more details on set_1, see Supporting information. In the validation, we screened set_1, followed by the statistical evaluation of the results obtained thereof with the Kruskal–Wallis test. Furthermore, we included in this analysis Bonferroni’s *post hoc* test, employing the confirmed inactive molecules in the *post hoc* test as control group, and accounting the results of this evaluation significant with *p *<0.1.

Additionally, we assembled set_2 by combining highly active and medium active inhibitors of set_1, resulting in 28 active molecules, and a virtual library of 12,775 putatively inactive molecules (‘decoys’).^44,45^ Afterwards, the virtual screening results from set_2 were used to calculate the percent yield (%*Y*) and the goodness of hit-list (GH)-score, which follow Eqs. 1 and 2. Furthermore, the enrichment factor (EF, Eq. 3) was calculated to compare the enrichment in the *x*% of the ranked hit-list to the ratio of actives to decoys in the entire validation set. We calculated the EF_1%_ and the EF_0.5%_ which may attain a maximal value of 100 or 200, respectively.^46^

(1) $$\%Y=\frac{\text{TP}}{n}*100$$

(2) $$\text{GH}=\left(\frac{\text{TP}*(3A+n)}{4\mathrm{nA}}\right)*\left(1-\frac{n-\text{TP}}{N-A}\right)$$

(3) $${\text{EF}}_{x\%}=\frac{{\text{TP}}_{x\%}/n_{x\%}}{A/N}$$

TP true positives, active molecules retained in the hit-list.
*n* number of hits found by the method.
*A* actives, all active molecules.
*N* all molecules, active molecules and the decoy set.

### 2.4 Forward filtering

First, to evaluate the enrichment obtained by employing 2D similarity screening, set_2 was utilized for virtual screening with 2D fingerprints. Later, in prospective virtual library screening 2D fingerprints were applied with adjusted and optimized settings and further filters: (i) a filter to focus on molecules with aqueous solubility level ⩾2, and (ii) Veber rules^47^ and Lipinski’s Rule-of-5.^48^ These filters were applied by performing respective protocols (‘ADMET Descriptors’ and ‘Filter by Lipinski and Veber Rules’) with default settings within PipelinePilot, while 2D similarity screening was performed within Discovery Studio with the protocol ‘Find Similar Molecules by Fingerprints’. The 2D similarity screening was performed with SciTegic fingerprints, representing a type of combinatorial/circular fingerprints.^49,50^ In the virtual screening campaign, the Vitas-M library was filtered which was downloaded in version September 2013 (http://www.vitasmlab.com/, 1,305,485 entries).

### 2.5 Conformational analysis

Prior to the hypotheses generation process, the conformational model of the training set compounds was generated using Discovery Studio with the more exhaustive ‘BEST’ quality^51^ and a maximum number of 255 conformations per molecule.

All compound libraries used for validating the pharmacophore models and in the prospective virtual library screening were converted into 3D multi-conformational databases using ‘CAESER’ quality^52^ with a maximum number of 100 conformations per molecule.

### 2.6 Pharmacophore modeling and virtual screening

The 3D pharmacophore models were generated employing the HipHop algorithm within Discovery Studio, which is available as protocol ‘Common Feature Pharmacophore Generation’. This algorithm elucidates the pharmacophore hypotheses in a so-called ‘pruned exhaustive search’. All two feature hypotheses, which are feasible in 3D space and are defined by the molecules in the training set as well as respective conformers as input, are generated. This is followed by the generation of more complex models utilizing the hypotheses retrieved in the previous step. The procedure is stopped when the generation process is exhausted, and then the best-ranked hypotheses are reported.^53,54^ Before hypotheses are created in a running, so-called principal values have to be assigned, to imply the influence a training set molecule has on the hypothesis generation process. Furthermore, the value for ‘Maximum Omitted Features’ (MOF) of a training set compound may be adjusted; when the value is set to 1, this implies that a ‘partial fit’ of this training set molecule is allowed. Additionally, the chemical feature types used for model building were set to include hydrogen-bond donor (HBD), hydrogen-bond acceptor (HBA), hydrophobic interaction (HYD), aromatic ring (RA), hydrophobic aromatic, and hydrophobic aliphatic (Hal).

Furthermore, pharmacophore-based virtual screening was performed by applying the ‘best flexible search’ algorithm. In this algorithm, the database entries are pre-filtered according to the absence or presence of all required chemical features needed for pharmacophore fitting in a rapid procedure. This is followed by a more rigorous procedure attempting to match the atoms of the 3D database molecule conformations to the features of the 3D search query. During the latter, small modifications of molecule’s conformation are allowed to enable and optimize the fitting of the molecule into the model. As limit for the optimized fitting into the model, the molecule’s strain energy is accounted during this procedure.^55^

### 2.7 Diversity clustering

To enhance the chemical diversity among the fitting molecules, diversity clustering was performed within Discovery Studio by applying the protocol ‘Cluster Ligands’ and using default settings to return 500 clusters. We collected the ‘cluster centers’ and submitted these compounds to molecular docking.

### 2.8 Molecular docking

Docking in the context of prospective virtual library screening was conducted employing Glide within the graphical interface, the Maestro suite, and as described previously.^56^ Following the preparation of the protein and the organic molecules, molecular docking was performed and hit-list ranking was achieved employing Glide in ‘standard precision’ (SP) mode.

For the binding pose predictions, the recently reported X-ray crystal structure of mPGES-1 with the co-crystallized ligand (PDB code: LVJ; PDB accession code: 4bpm)^36^ was utilized for the docking study. The macromolecule 3D coordinates were downloaded from the PDBe^57^ providing a web portal (http://www.ebi.ac.uk/pdbe/). We employed LigandScout^58^ to deduce critical molecular interactions as 3D pharmacophore as well as for visualization purposes.

### 2.9 Induction of mPGES-1 in A549 cells, isolation of microsomes, and determination of mPGES-1 activity

Human A549 cells were treated and prepared as described.^59^ Briefly, cells (2 × 10^6^/20 ml DMEM/High glucose (4.5 g/L) medium containing FCS (2%, v/v)) were incubated for 16 h at 37 °C, 5% CO_2_. The culture medium was replaced by fresh medium, interleukin-1β (1 ng/ml) was added, and cells were incubated for another 72 h. Then, cells were detached with trypsin/EDTA, washed with PBS and frozen in liquid nitrogen. Ice-cold homogenization buffer (0.1 M potassium phosphate buffer pH 7.4, 1 mM phenylmethylsulfonyl fluoride, 60 μg/ml soybean trypsin inhibitor, 1 μg/ml leupeptin, 2.5 mM glutathione, and 250 mM sucrose) was added. After 15 min on ice, cells were resuspended and sonicated (3 × 20 s). The homogenate was subjected to differential centrifugation (10,000×*g* for 10 min and at 174,000×*g* for 1 h at 4 °C). The pellet (microsomal fraction) was resuspended in 1 ml homogenization buffer, and protein concentration was determined by the Coomassie protein assay. The microsomal membranes were then diluted in potassium phosphate buffer (0.1 M, pH 7.4) containing 2.5 mM glutathione (100 μl total volume) and test compounds or vehicle (DMSO) were added. After 15 min, enzymatic PGE_2_ formation was initiated by addition of 20 μM PGH_2_ (final concentration). After 1 min at 4 °C, the reaction was terminated with 100 μl of stop solution (40 mM FeCl_2_, 80 mM citric acid and 10 μM of 11β-PGE_2_), PGE_2_ was separated by solid phase extraction on reversed phase (RP)-C18 material using acetonitrile (200 μl) as eluent, and analyzed by RP-HPLC (30% acetonitrile aqueous + 0.007% TFA (v/v), Nova-Pak® C18 column, 5 × 100 mm, 4 μm particle size, flow rate 1 ml/min) with UV detection at 195 nm. 11β-PGE_2_ was used as internal standard to quantify PGE_2_ formation.

## 3 Results and discussion

### 3.1 Validation of the 2D search query

**Figure 3:**
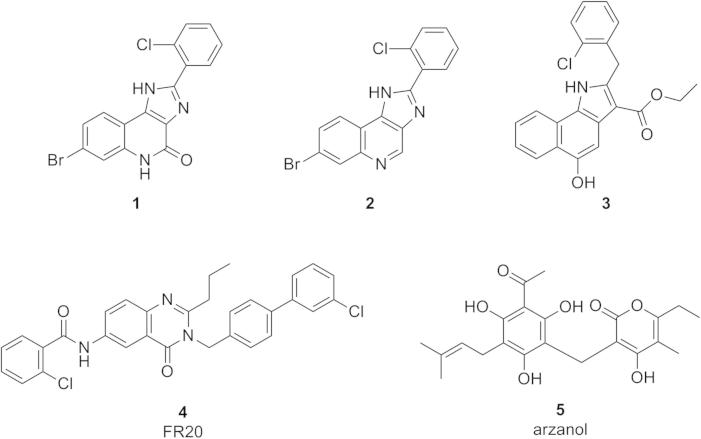
Training set compounds for pharmacophore modeling.

In brief, various runs showed that the best results were achieved with ECFP4 fingerprints with a Tanimoto coefficient ⩾0.25 and a multiple-molecule query consisting of compounds **1**, **3**, and **4**, which represented the most potent and chemically diverse molecules also later employed in the creation of the pharmacophore model (Fig. 3).^20,25,26^ In this combination, a high enrichment was achieved in the screening experiments using set_2 (%*Y* = 48.28, GH = 0.49, EF_1%_ = 67.87, EF_0.5%_ = 121.46).

### 3.2 Pharmacophore model validation

In order to select the best-performing model for the virtual screening campaign, the hypotheses were validated thoroughly. The synthetic compounds **1**–**4** and the natural product arzanol (**5**) from the medicinal plant *Helichrysum italicum* were selected as training set (Fig. 3) for pharmacophore modeling.^20,25,26,60^ We assigned compound **1** a principal value of 2 (highly active), while the remaining compounds in the training set **2**–**5** were assigned a principal value of 1 (active, Table 1). Furthermore, a different binding mode was assumed in case of **5**, and so the value for MOF was adjusted to 1, while this value was left unmodified among the other compounds in the training set. This adjustment implies that a ‘partial fit’ of all compounds, except compound **5**, was not allowed during the hypothesis generation process.

**Figure 4:**
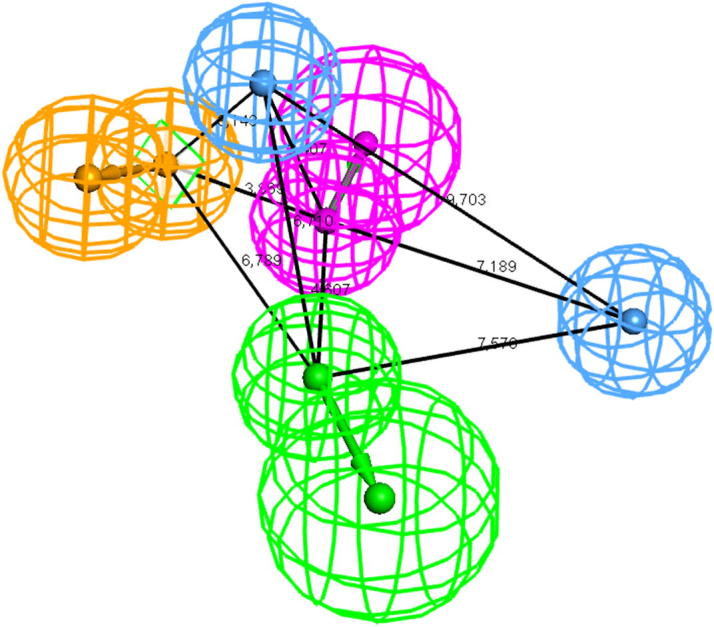
Depiction of Hypo01; chemical features are color-coded: light blue-Hal; orange-RA; magenta-HBD; green-HBA.

Among the 10 computed hypotheses, Hypo01 and Hypo06 showed the most promising results. They returned 35.7% of the highly active inhibitors, respectively, and 57.1% (Hypo01) or 50.0% (Hypo06) of the medium active inhibitors, while all confirmed inactive molecules were discarded. Accounting in the evaluation the Kruskal–Wallis’ statistic, good model quality was achieved in case of Hypo01 and Hypo06 (Table 2). The differences between these two most promising 3D pharmacophore models were quite subtle. Hypo01 consisted of one RA, one HBA, one HBD, and two Hal, while Hypo06 comprised basically the same features, apart from one HYD in the position of the RA. The statistical evaluation with the *post hoc* test showed, that in case of Hypo01, confirmed inactive molecules were separated from medium active inhibitors (*p* = 0.0029) and highly active inhibitors (*p* = 0.0523) by their geometrical fit score, displaying the best results among all hypotheses. The results from the screening experiments of set_1 with Hypo01 are available in Supporting information. In the benchmarking experiments, Hypo01 together with Hypo07 to Hypo10 showed the most promising results when the GH-score was accounted (GH ⩾0.15). The EF-values prioritized Hypo01 together with Hypo06 to Hypo10. Especially, when EF_0.5%_ was accounted, Hypo01 outperformed the other hypotheses, while Hypo01 also attained the best results in terms of%*Y*. Together, the results pointed towards superior model quality in case of Hypo01 (Table 2). Hypo01, the best-performing pharmacophore model is shown in Figure 4.

### 3.3 Virtual screening using an external library

**Figure 5:**
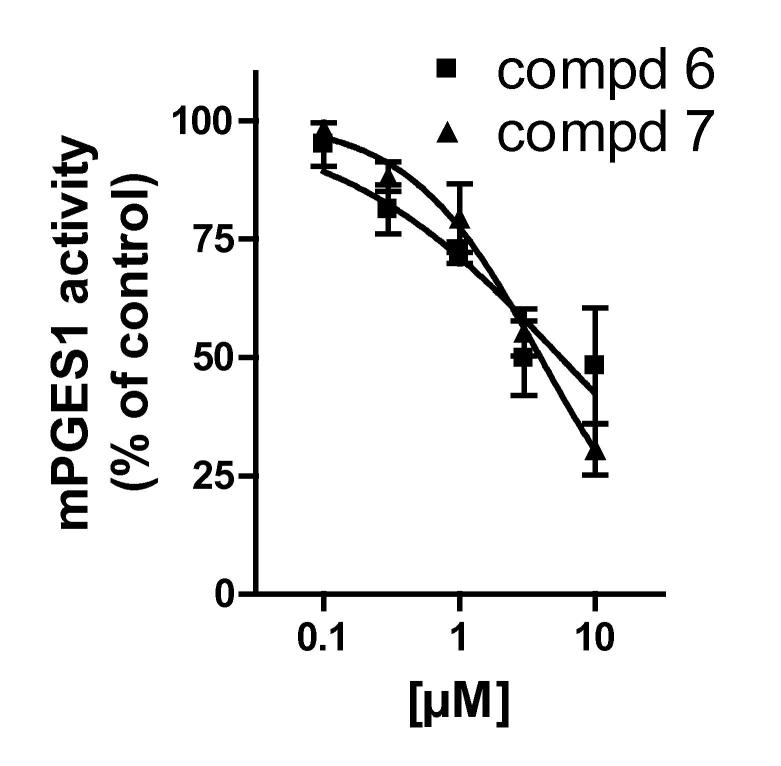
Inhibition of mPGES-1 by compounds **6** and **7**. Data are given as mean ± SEM; *n* = 3.

Finally, to experimentally validate the virtual screening protocol, the Vitas-M library (http://www.vitasmlab.com/) was filtered. Out of the initial compound library, which comprised 1,305,485 molecules, 1,020,417 molecules (78.2%) and 974,991 molecules (74.7%) were retrieved in the forward filtering by applying the filter on the aqueous solubility and the Lipinski and Veber rules, respectively. Then, out of the compound library which was returned from these previous filters, a focused library of 18,057 molecules (1.4%) was retrieved by the application of 2D similarity screening. Afterwards, pharmacophore-based virtual screening was performed returning 8079 fitting molecules (0.6% of the initial Vitas-M library). We only considered molecules for further processing, which attained high fit-values (pharmacophore fit ⩾2.5). This reduced the number of remaining molecules to 1857 (0.14% of the initial library). To enhance the structural diversity of the hits to be biologically tested, these molecules were clustered. The 500 molecules (0.04% of the Vitas-M collection), retrieved as cluster centers were submitted to molecular docking. After re-scoring by molecular docking, the hit-list was visually inspected. Finally, 20 molecules were selected of which 17 were available and acquired for biological evaluation in the cell-free mPGES-1 activity assay. Among the tested molecules (10 μM, final concentration), compounds **6** and **7** were revealed as efficient inhibitors of mPGES-1-mediated PGE_2_ synthesis (Table 3). Concentration–response analysis for compounds **6** and **7** revealed IC_50_ values of 4.5 and 3.8 μM (Fig. 5), potencies that are close to those of the reference inhibitor MK-886 (IC_50_ = 2.4 μM) in a comparable test system.^61^ In addition, compounds **8** and **9** significantly suppressed mPGES-1 activity at 10 μM, but less than 50% (Table 3). Thus, IC_50_ values were not determined. The other 13 molecules out of the 17 acquired ones (which are shown in Supporting information) failed to inhibit mPGES-1 at 10 μM or were not determined.

In order to confirm that the most potent compounds **6** and **7** are specific for mPGES-1, we tested on one hand if reduced mPGES-1 activity is simply due to irreversible or nuisance inhibition, and on the other hand, we analyzed if they also affect related enzymes within the AA cascade. Wash-out experiments revealed reversible mPGES-1 inhibition (Fig. S1), and triton-X100 failed to abolish the mPGES-1 inhibitory activity of **6** and **7** (Fig. S2), thus, excluding unspecific interference. Moreover, **6** and **7** were poor inhibitors of isolated COX-2 (IC_50_ >10 μM, Fig. S3). However, **7** effectively inhibited 5-lipoxygenase in a cell-free assay with an IC_50_ value of about 5 μM, whereas **6** was less active (IC_50_ >10 μM, Fig. S4). Note that dual inhibition of mPGES-1 and 5-lipoxygenase is common to many structural classes of inhibitors of natural synthetic origin.^62^ Together, we conclude that **6** and **7** rather specifically interact with mPGES-1 and inhibit its activity.

**Figure 6:**
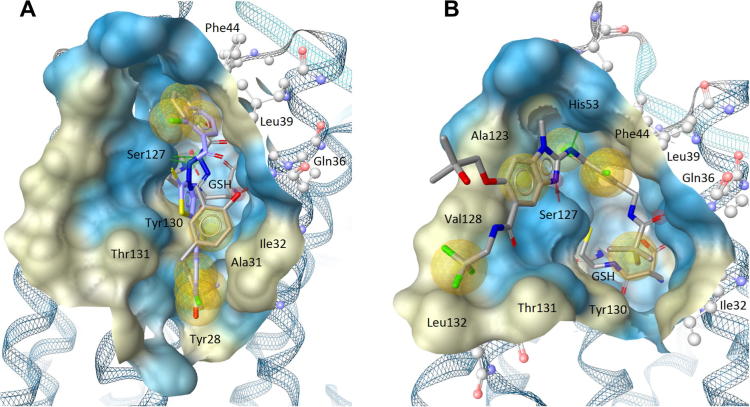
(A) Predicted binding modes, shown for the two most active molecules yielded in the virtual screening campaign, compounds **6** (gray) and **7** (magenta). (B) In comparison, the binding mode of the highly potent inhibitor LVJ is depicted, following the minimization and 3D pharmacophore creation within LigandScout. Glutathione (GSH); chemical-features are color-coded: red arrow—HBA; green arrow—HBD, yellow sphere—HYD; the poses are shown with receptor-binding surface (color-coded by aggregated hydrophilicity/hydrophobicity: blue/gray, respectively).

To obtain a more profound insight on potential binding modes, the virtual screening hits were submitted to molecular docking. Accounting the docking poses of compounds **6** and **7** (Fig. 6A), these novel bioactive molecules were predicted to be involved in hydrogen-bonding to Ser127, which is assumed to serve as a key residue in the catalytic activity.^35^ Furthermore, a hydrogen-bond was predicted to be formed between **6** and Gln36. Interestingly, both inhibitors were predicted to adopt a conformation, where these ligands complement the surface of the binding site adjacent to glutathione. In case of **6**, a substituted benzene ring was predicted to be orientated close to Phe44 and Leu39, which formed a hydrophobic contact to this ligand moiety. In case of **7**, the benzene ring, which was predicted to bind next to Phe44, has a chlorine substituent attached, which could be involved in a hydrophobic interaction with Leu39. The substituted benzene moieties of **6** and **7**, which were oriented towards the opposite site of the mPGES-1 binding site, were predicted to be embedded in a hydrophobic site formed by mainly aromatic or hydrophobic amino acids (e.g., Tyr28, Tyr130, Thr131, Ala31, and Ile32). In comparison, LVJ (Fig. 6B), which has inhibitory potency on mPGES-1 activity in the low nanomolar range, adopts a position and orientation in the binding pocket which is slightly shifted towards mainly hydrophobic amino acids (e.g., Ala123, Val128, and Leu132), while several hydrogen-bonds are formed to the key residue Ser127 and other residues (e.g., Gln36 and His53).

## 4 Discussion

We herein report the discovery of four novel molecules suppressing mPGES-1 activity, of which the two most active ones showed the desired activity in the low micromolar range. When only regarding the most active compounds, a hit rate of 12.5% (two virtual screening hits out of 16 tested molecules) was achieved. Interestingly, this is comparable to other studies, in which prospective virtual library screening for the discovery of novel and non-acidic mPGES-1 inhibitors was conducted. For instance, He et al. attained good results by employing a molecular dynamics simulation to obtain an altered conformation of the 3D-structure of mPGES-1, which was modified towards an active state conformation and utilized in a docking-based screening strategy. Following the in silico approach, 21 molecules of 142 tested molecules showed the desired activity in the experimental evaluation (hit rate: 14.8%).^32^ Furthermore, Rörsch et al. applied basically ligand-based methods in the search for mPGES-1 inhibitors. Following the experimental evaluation of 17 molecules, three novel bioactive molecules were discovered and for one of those an IC_50_ value was determined, showing that this compound exerts potency in the sub-micromolar range.^29^ Fortunately, very recently the 3D-structure of mPGES-1 with a co-crystallized inhibitor became available.^36^ We therefore utilized this 3D-structure in a docking study of the two most active molecules, yielded in this study, compounds **6** and **7**, in order to surmise binding modes of these novel mPGES-1 inhibitors. We thereby predicted that these molecules are accommodated nicely in the site adjacent to the cofactor glutathione, and could exhibit molecular interactions to the key residue Ser127. Together, the compounds **6** and **7** showed inhibitory potency on mPGES-1 activity in the low micromolar range, making them interesting starting points for optimization efforts.

Basically, virtual screening techniques are usually validated by screening (a) validation set(s) in benchmarking experiments. In cases like this study, where very similar models perform with quite comparable results, an additional validation with the Kruskal–Wallis test can be helpful in the selection of the screening model, especially as this test is considered to serve as robust investigation of the model quality.^38,39^

## 5 Conclusion

In summary, a multistep virtual screening protocol is presented, which included a novel concept in the validation of the 3D pharmacophore. Following a virtual screening campaign the results of the experimental evaluation confirmed the protocol quality, while the two most active molecules which inhibited mPGES-1 in a cell-free mPGES-1 activity assay, compounds **6** and **7**, may serve as promising starting points for further optimization. The results may be considered as a case study; however, the modified concept applied in the pharmacophore model validation may be useful for further studies on other targets.

## Figures and Tables

**Table 1:** Training set for HipHop model

Compound	IC_50_ (μM)	Class	Reference
**1**	0.0091	Highly active	26
**2**	0.25	Active	26
**3**	0.1	Active	20
**4**	0.13	Active	25
**5**	0.4	Active	60

**Table 2:** Results from the theoretical validation of the 3D pharmacophore models

	%*Y*	GH	EF_1%_	EF_0.5%_	Kruskal–Wallis’ statistic	*p*
Hypo01	4.45	0.15	32.14	50.00	9.78	0.0075
Hypo02	1.54	0.12	17.86	35.71	5.04	0.0806
Hypo03	1.55	0.12	17.86	35.71	5.65	0.0593
Hypo04	0.99	0.10	14.29	21.43	5.30	0.0705
Hypo05	0.99	0.10	14.29	14.29	5.30	0.0705
Hypo06	2.36	0.12	25.00	35.71	8.36	0.0153
Hypo07	2.58	0.15	28.57	35.71	5.37	0.0682
Hypo08	1.37	0.16	21.43	42.86	4.89	0.0869
Hypo09	1.58	0.15	39.29	35.71	3.74	0.1538
Hypo10	1.61	0.15	32.14	28.57	2.99	0.2238

**Table 3:** Summary of results from the experimental evaluation for the four novel molecules, suppressing mPGES-1 activity, among the 16 tested molecules

Compound	Chemical structure	Remaining activity at 10 μM (%)	IC_50_ (μM)
**6**		32.78 ± 1.73	4.5
**7**		34.22 ± 2.56	3.8
**8**		63.8 ± 6.21	—
**9**		64.0 ± 2.30	—
