# Supplementary material for: Discovery of novel, non-acidic mPGES-1 inhibitors by virtual screening with a multistep protocol
Source: Bioorg Med Chem. 2015 Aug 1;23(15):4839–45. doi: 10.1016/j.bmc.2015.05.045 (PMC4528062; doi:10.1016/j.bmc.2015.05.045)
Supplement: Supplementary data — Supplementary material. [file mmc1.pdf]

Supporting Information:

Discovery of novel, non-acidic mPGES-1 inhibitors by virtual screening with a multistep protocol

Stefan M. Noha, Katrin Fischer, Andreas Koeberle, Ulrike Garscha, Oliver Werz and Daniela Schuster

This supporting material document contains

1.) Overview of set_1 assembled from nine chemical series of non-acidic mPGES-1 inhibitors (Table S1)
2.) 2D structures of organic molecules of set_1 which were assembled accounting pre-defined ranges for the biological activity of respective molecules (Charts S1-S3)
3.) Detailed results on virtual screening experiments of set_1 with Hypo01 (Table S2-S4)
4.) Molecules tested in the biological evaluation and which did not show the desired activity (Chart S4 and Table S5)
5.) Biologicals assays: cell-free assays for 5-lipoxygenase and cyclooxygenase-2 activity
6.) Biological evaluation (Supplemental Figures S1-S4)

1.) <u>Overview of set_1 assembled from nine chemical series of non-acidic mPGES-1 inhibitors</u>

Table S1. Set_1 composition overview.

Chemical scaffold	Highly active inhibitors	Medium active inhibitors	Confirmed inactive molecules
quinazolinones[1]	2	0	3
imidazol-2-yl-benzamids[2]	1	0	0
carbazol-3-yl-benzamid (AF3442)[3]	1	0	0
biaryl imidazoles[4]	2	4	1
phenanthrene imidazoles[5-6]	2	2	3
benzo[g]indol-3-carboxylates[7]	2	3	2
imidazoquinolines[8]	2	3	3
trisubstituted ureas[9]	2	1	2
benzoxazoles[10]	0	1	0

2.) <u>2D structures of organic molecules of set_1 which were assembled accounting pre-defined ranges for the biological activity of respective molecules</u>

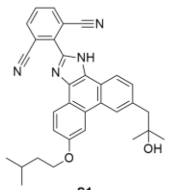

S1
IC_{50} = 0.0004 µM

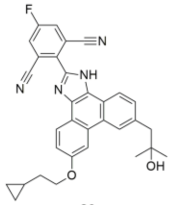

S2
IC_{50} = 0.001 µM

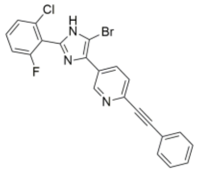

S3
IC_{50} = 0.001 µM

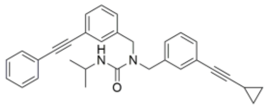

S4
IC_{50} = 0.001 µM

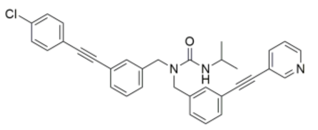

S5
IC_{50} = 0.001 µM

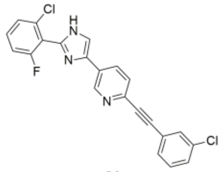

S6
IC_{50} = 0.004 µM

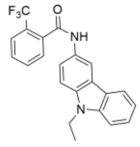

S7
IC_{50} = 0.06 µM

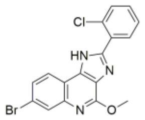

S8
IC_{50} = 0.06 µM

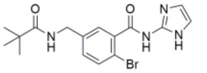

S9
IC_{50} = 0.18 µM

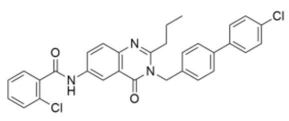

S10
IC_{50} = 0.18 µM

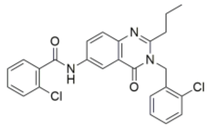

S11
IC_{50} = 0.18 µM

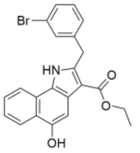

S12
IC_{50} = 0.2 µM

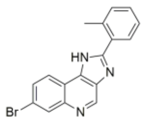

S13
IC_{50} = 0.395 µM

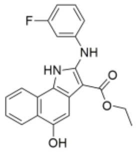

S14
IC_{50} = 0.5 µM

Chart S1. Highly active inhibitors of set_1, assembled from the congeneric series of non-acidic mPGES-1 inhibitors (IC_{50} ≤ 0.5 µM).

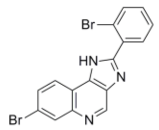

S15
IC_{50} = 0.506 μM

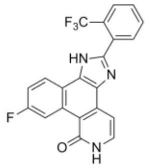

S16
IC_{50} = 0.56 μM

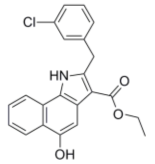

S17
IC_{50} = 0.6 μM

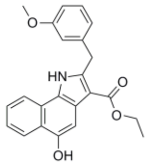

S18
IC_{50} = 0.6 μM

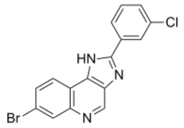

S19
IC_{50} = 0.9 μM

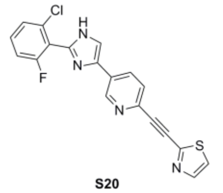

S20
IC_{50} = 0.94 μM

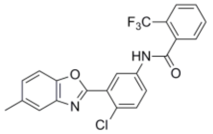

S21
IC_{50} = 1.3 μM

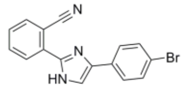

S22
IC_{50} = 1.4 μM

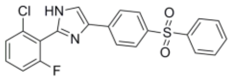

S23
IC_{50} = 1.4 μM

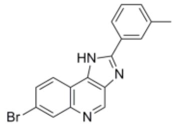

S24
IC_{50} = 1.5 μM

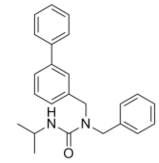

S25
IC_{50} = 1.7 μM

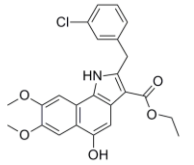

S26
IC_{50} = 1.7 μM

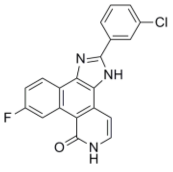

S27
IC_{50} = 2.5 μM

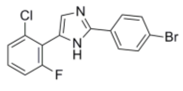

S28
IC_{50} = 3.7 μM

Chart S2. Medium active inhibitors of set_1, assembled from the congeneric series of non-acidic mPGES-1 inhibitors (IC_{50}: 0.5 – 5 μM).

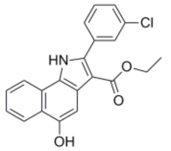

S29
IC_{50} = 9.2 µM

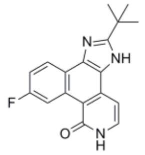

S30
IC_{50} > 10 µM

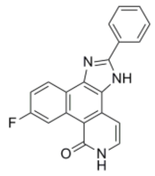

S31
IC_{50} > 10 µM

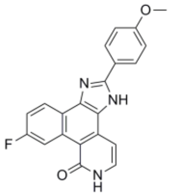

S32
IC_{50} > 10 µM

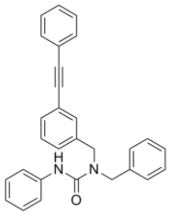

S33
IC_{50} > 10 µM

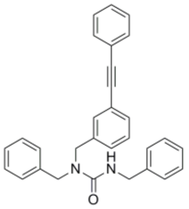

S34
IC_{50} > 10 µM

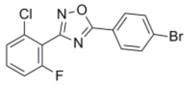

S35
IC_{50} > 30 µM

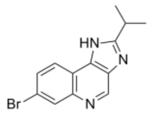

S36
i.a.

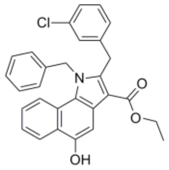

S37
i.a.

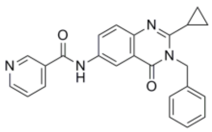

S38
i.a.

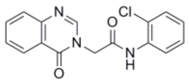

S39
i.a.

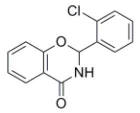

S40
i.a.

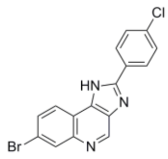

S41
i.a.

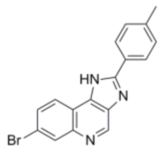

S42
i.a.

Chart S3. Confirmed inactive molecules of set_1, assembled from the congeneric series of non-acidic mPGES-1 inhibitors (IC_{50} > 5 µM).

3.) Detailed results on virtual screening experiments of set_1 with Hypo01

Table S2. Highly active inhibitors of set_1 with fit-values attained by virtual screening experiments with Hypo01.

Compound	Fit-value	IC_{50} [µM]	Chemical scaffold
S1	--	0.0004	phenanthrene imidazoles
S2	--	0.001	phenanthrene imidazoles
S3	--	0.001	biaryl imidazoles
S4	--	0.001	trisubstituted ureas
S5	--	0.001	trisubstituted ureas
S6	--	0.004	biaryl imidazoles
S7	1.99465	0.06	carbazol-3-yl-benzamid (AF3442)
S8	4.52286	0.06	imidazoquinolines
S9	0.879768	0.18	imidazol-2-yl-benzamids
S10	2.3017	0.18	quinazolinones
S11	3.43283	0.18	quinazolinones
S12	--	0.2	benzo[g]indol-3-carboxylates
S13	--	0.395	imidazoquinolines
S14	--	0.5	benzo[g]indol-3-carboxylates

Table S3. Medium active inhibitors of set_1 with fit-values attained by virtual screening experiments with Hypo01.

Compound	Fit-value	IC_{50} [µM]	Chemical scaffold
S15	4.11122	0.506	imidazoquinolines
S16	--	0.56	phenanthrene imidazoles
S17	1.062	0.6	benzo[g]indol-3-carboxylates
S18	0.91741	0.6	benzo[g]indol-3-carboxylates
S19	1.68216	0.9	imidazoquinolines
S20	--	0.94	biaryl imidazoles
S21	1.49908	1.3	benzoxazoles
S22	--	1.3	biaryl imidazoles
S23	--	1.4	biaryl imidazoles
S24	--	1.5	imidazoquinolines
S25	--	1.7	trisubstituted ureas
S26	3.37656	1.7	benzo[g]indol-3-carboxylates
S27	0.210956	2.5	phenanthrene imidazoles
S28	3.95012	3.7	biaryl imidazoles

Table S4. Confirmed inactive molecules of set_1 with fit-values attained by virtual screening experiments with Hypo01.

Compound	Fit-value	IC_{50} [µM]	Chemical scaffold
S29	--	9.2	benzo[g]indol-3-carboxylates
S30	--	>10	phenanthrene imidazoles
S31	--	>10	phenanthrene imidazoles
S32	--	>10	phenanthrene imidazoles
S33	--	>10	trisubstituted ureas
S34	--	>10	trisubstituted ureas
S35	--	>30	biaryl imidazoles
S36	--	i.a.[a]	imidazoquinolines
S37	--	i.a.	benzo[g]indol-3-carboxylates
S38	--	i.a.	quinazolinones
S39	--	i.a.	quinazolinones
S40	--	i.a.	quinazolinones
S41	--	i.a.	imidazoquinolines
S42	--	i.a.	imidazoquinolines

[a] i.a. = inactive.

4.) <u>Molecules tested in the biological evaluation and which did not show the desired activity</u>

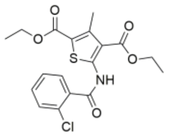

S43

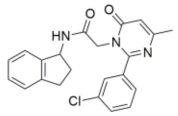

S44

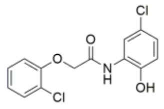

S45

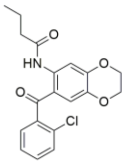

S46

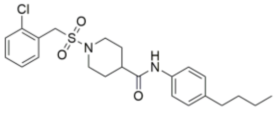

S47

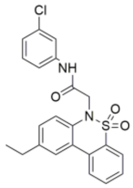

S48

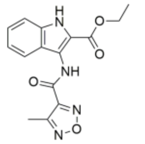

S49

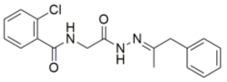

S50

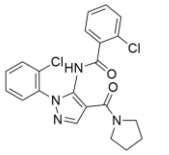

S51

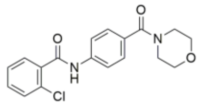

S52

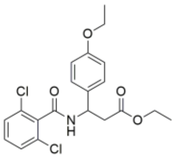

S53

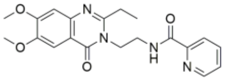

S54

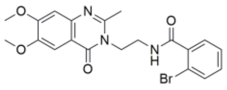

S55

Chart S4. Remaining compounds not showing the desired activity are depicted with 2D structures.

Table S5. Remaining mPGES-1 activity (%) at a final concentration of 10 µM ± SEM.

Compound	Remaining activity at 10 µM (% ± SEM)
**S43**	86.5 ± 5.83
**S44**	94.7 ± 1.88
**S45**	95.6 ± 6.82
**S46**	82.8 ± 5.77
**S47**	93.4 ± 6.09
**S48**	85.0 ± 6.92
**S49**	88.3 ± 4.56
**S50**	88.4 ± 3.18
**S51**	–
**S52**	94.2 ± 1.87
**S53**	91.5 ± 3.97
**S54**	90.0 ± 5.80
**S55**	94.8 ± 10.14

5.) Biologicals assays: cell-free assays for 5-lipoxygenase and cyclooxygenase-2 activity

Activity assays of isolated COX-2

Purified COX-2 (human recombinant, 20 units) was diluted in 1 mL Tris buffer (100 mM) pH 8, containing 5 mM glutathione, 5 µM hemoglobin, and 100 µM EDTA at 4 °C and pre-incubated with the test compound for 5 min. Samples were pre-warmed for 60 s at 37 °C, and 2 µM AA was added. After 5 min at 37 °C, the reaction was stopped, PGB_1 as standard added and the COX product 12-hydroxy-5,8,10-heptadecatrienoic acid (12-HHT) was extracted and then analyzed by HPLC.

Determination of 5-lipoxygenase activity in cell-free systems

E.coli BL21 was transformed with pT3-5LO plasmid, human recombinant 5-lipoxygenase protein was expressed at 37 °C, purified, and assayed as described.[11] In brief, purified 5-lipoxygenase (0.5 µg) was diluted with PBS pH 7.4 plus 1 mM EDTA and pre-incubated with the test compounds. After 15 min at 4 °C, samples were pre-warmed for 30 s at 37 °C, and 2 mM $CaCl_2$ plus 20 µM AA were added. After 10 min at 37 °C formed 5-lipoxygenase metabolites were analyzed by HPLC as described.[11]

6.) Biological evaluation (Supplemental Figures S1-S4)

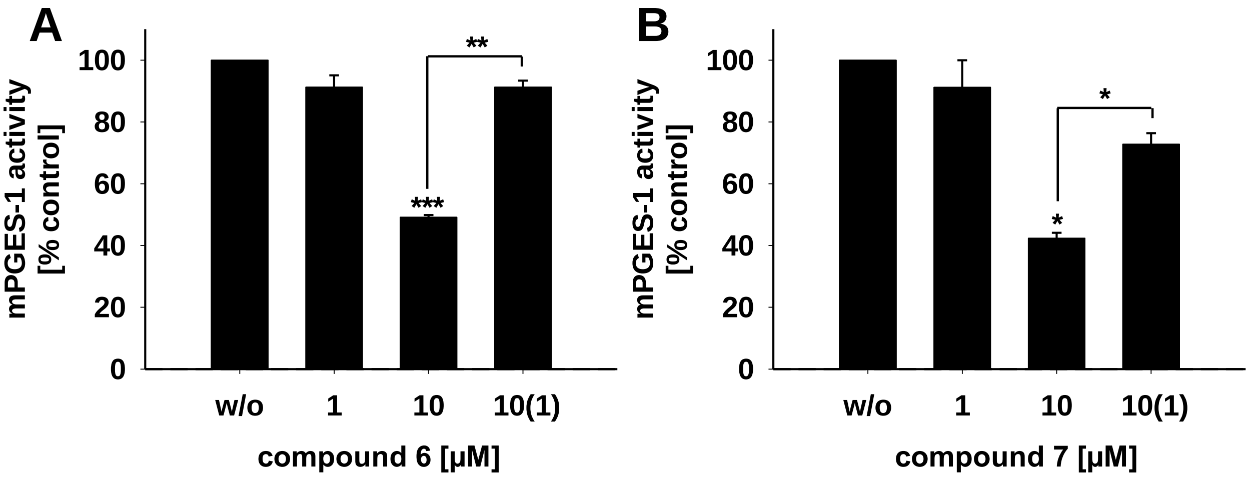

Figure S1. Reversibility of mPGES-1 inhibition. (**A**, **B**) Microsomal preparations of interleukin-1β-stimulated A549 cells were pre-incubated with 10 µM compound **6** (**A**) or **7 (B)** for 15 min at 4°C and then diluted 10-fold to obtain an inhibitor concentration of 1 µM. For comparison, microsomal preparations were pre-incubated with 1 or 10 µM compound and then diluted 10-fold while maintaining the inhibitor concentration. All samples were incubated on ice for 1 min, and PGE_2 was analyzed by HPLC. Data are given as mean ± S.E. of single determinations obtained in three independent experiments. (*) P <0.05, (**) P <0.01, (***) P <0.001; ANOVA + Tukey HSD *post-hoc* tests.

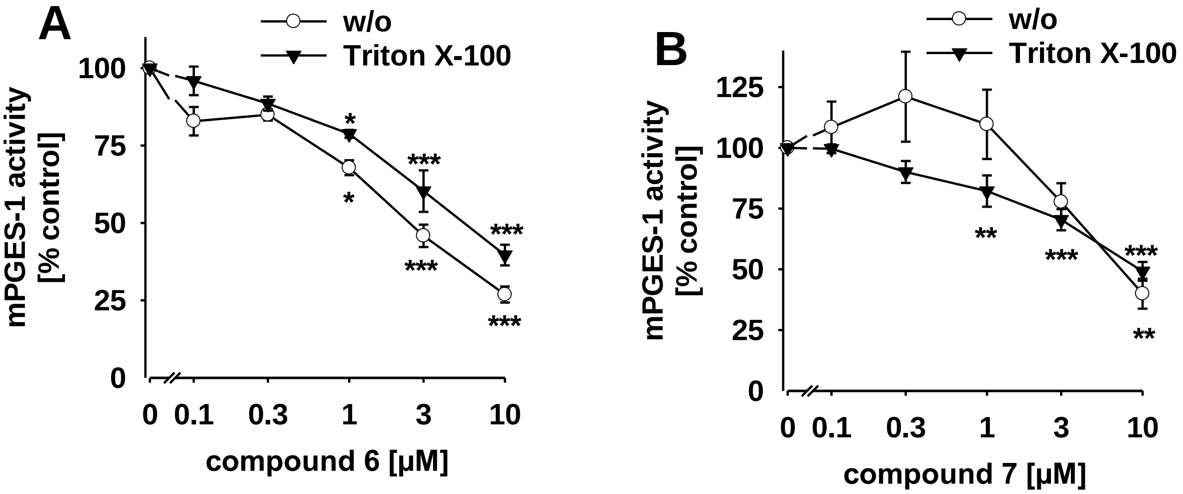

Figure S2. Nuisance inhibition of mPGES-1. (**A**, **B**) The effect of compound **6** (**A**) and **7** (**B**) on mPGES-1 activity was determined in absence and presence of triton X-100 (0.01%, v/v). Data are given as mean ± S.E. of single determinations obtained in three independent experiments. (*) *P* <0.05, (**) *P* <0.01, (***) *P* <0.001; ANOVA + Tukey HSD *post-hoc* tests.

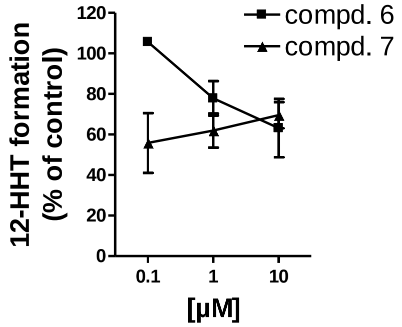

Figure S3. Effects of compounds **6** and **7** on the activity of COX-2 in a cell-free assay. Data are given as mean ± S.E. of single determinations obtained in three independent experiments.

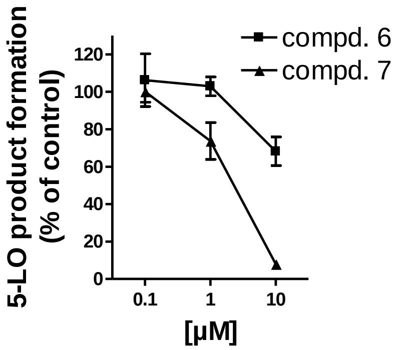

Figure S4. Effects of compounds **6** and **7** on the activity of 5-lipoxyenase in a cell-free assay. Data are given as mean ± S.E. of single determinations obtained in three independent experiments.

References

1. Rorsch, F.; Buscato, E.; Deckmann, K.; Schneider, G.; Schubert-Zsilavecz, M.; Geisslinger, G.; Proschak, E.; Grosch, S. *J. Med. Chem.* **2012**, *55*, 3792.
2. Abdel-Magid, A. F. *ACS Medicinal Chemistry Letters* **2012**, *3*, 703.
3. Bruno, A.; Di Francesco, L.; Coletta, I.; Mangano, G.; Alisi, M. A.; Polenzani, L.; Milanese, C.; Anzellotti, P.; Ricciotti, E.; Dovizio, M.; Di Francesco, A.; Tacconelli, S.; Capone, M. L.; Patrignani, P. *Biochem. Pharmacol.* **2010**, *79*, 974.
4. Wu, T. Y.; Juteau, H.; Ducharme, Y.; Friesen, R. W.; Guiral, S.; Dufresne, L.; Poirier, H.; Salem, M.; Riendeau, D.; Mancini, J.; Brideau, C. *Bioorg. Med. Chem. Lett.* **2010**, *20*, 6978.
5. Cote, B.; Boulet, L.; Brideau, C.; Claveau, D.; Ethier, D.; Frenette, R.; Gagnon, M.; Giroux, A.; Guay, J.; Guiral, S.; Mancini, J.; Martins, E.; Masse, F.; Methot, N.; Riendeau, D.; Rubin, J.; Xu, D.; Yu, H.; Ducharme, Y.; Friesen, R. W. *Bioorg. Med. Chem. Lett.* **2007**, *17*, 6816.
6. Giroux, A.; Boulet, L.; Brideau, C.; Chau, A.; Claveau, D.; Cote, B.; Ethier, D.; Frenette, R.; Gagnon, M.; Guay, J.; Guiral, S.; Mancini, J.; Martins, E.; Masse, F.; Methot, N.; Riendeau, D.; Rubin, J.; Xu, D.; Yu, H.; Ducharme, Y.; Friesen, R. W. *Bioorg. Med. Chem. Lett.* **2009**, *19*, 5837.
7. Koeberle, A.; Haberl, E. M.; Rossi, A.; Pergola, C.; Dehm, F.; Northoff, H.; Troschuetz, R.; Sautebin, L.; Werz, O. *Bioorg. Med. Chem.* **2009**, *17*, 7924.
8. Shiro, T.; Takahashi, H.; Kakiguchi, K.; Inoue, Y.; Masuda, K.; Nagata, H.; Tobe, M. *Bioorg. Med. Chem. Lett.* **2012**, *22*, 285.
9. Chiasson, J. F.; Boulet, L.; Brideau, C.; Chau, A.; Claveau, D.; Cote, B.; Ethier, D.; Giroux, A.; Guay, J.; Guiral, S.; Mancini, J.; Masse, F.; Methot, N.; Riendeau, D.; Roy, P.; Rubin, J.; Xu, D.; Yu, H.; Ducharme, Y.; Friesen, R. W. *Bioorg. Med. Chem. Lett.* **2011**, *21*, 1488.
10. Pelcman, B.; Olofsson, K.; Schaal, W.; Kalvins, I.; Katkevics, M.; Ozola, V.; Suna, E. PCT Int. Appl. WO 2007042816, **2007**.
11. Pergola, C.; Jazzar, B.; Rossi, A.; Northoff, H.; Hamburger, M.; Sautebin, L.; Werz, O. *Br. J. Pharmacol.* **2012**, *165*, 765.
